# 

**Published:** 1936

**Authors:** 


### The Bristol Medico-Chirurgical Journal

A JOURNAL OF THE MEDICAL SCIENCES FOR THE WEST OF ENGLAND AND SOUTH WALES.

PUBLISHED UNDER THE AUSPICES OF

*THE BRISTOL MEDICO-CHIRURGICAL SOCIETY.*

EDITOR :

J. A. NIXON, C.M.G., M.D., F.R.C.P.

WITH WHOM ARE ASSOCIATED

E. W. HEY GROVES, D.Sc., M.S., F.R.C.S.
A. E. ILES, O.B.E., M.B., B.S., F.R.C.S.
A. RENDLE SHORT, M.D., B.S., B.Sc., F.R.C.S.
E. WATSON-WILLIAMS, M.C., Ch.M., F.R.C.S., Assistant Editor.

EDITORIAL SECRETARY :

A. L. FLEMMING, M.B., Ch.B.

---

"Scire est nescire, nisi id me

Scire alius sciret."

---

*VOL. LIII.*

ARMY MEDICAL LIBRARY

Sept. 15, 1938

326768

WASHINGTON, D.C., U.S.A.

BRISTOL: J. W. ARROWSMITH LTD.
London: J. W. Arrowsmith (London) Ltd.

WI
BR323

### Contents of Vol. LIII

| | Page |
|---|---|
| In Memoriam: King George V. .. .. .. .. .. .. | 1 |
| Photography and Medicine. By A. E. Iles, O.B.E., M.B., F.R.C.S. | 3 |
| Ultra-Microscopic Examination of the Blood-Serum in Disease. By B. A. Peters, M.D., D.P.H. .. .. .. .. .. .. | 17 |
| Bristol Eye Hospital. By The Editor .. .. .. .. .. | 35 |
| The Position of the Voluntary Hospitals in the National Health Services. By Robert F. Barclay, LL.D. .. .. .. .. .. | 39 |
| Corneal Transplantation: Its History, Development and Practice. By J. W. Tudor Thomas, D.Sc., M.D., M.S., F.R.C.S. .. | 75 |
| A Treatment for Sciatica. By A. Rendle Short, M.D., B.S., B.Sc., F.R.C.S. .. .. .. .. .. .. .. .. .. | 87 |
| Carcinoma of the Bronchus:— | |
| Clinical Features. By C. Bruce Perry, M.D., F.R.C.P. .. .. | 125 |
| Primary Carcinoma of the Lung Radiologically Considered. By Gilbert B. Bush, M.B., Ch.B., D.M.R.E. .. .. .. | 131 |
| Bronchoscopic Diagnosis of Carcinoma of the Lung. By Gordon Scarff, M.B., Ch.B., F.R.C.S. .. .. .. .. .. | 137 |
| The Pathological Aspect. By Arthur L. Taylor, M.D. .. .. | 139 |
| The Treatment of Carcinoma of the Lung. By Duncan Wood, F.R.C.S. .. .. .. .. .. .. .. .. .. | 145 |
| A Fatal Case of Meningitis Due to Infection of the Sphencidal Sinus by Pfeiffer's Bacillus, with a Short Review of Methods of Treatment. By C. Elaine Field, M.B., B.S., and Herbert Rogers, M.D. | 151 |
| Surgical Clinics in Scandinavia. By A. Rendle Short, M.D., B.S., B.Sc., F.R.C.S. .. .. .. .. .. .. .. .. | 157 |
| Report of the Committee on the Scottish Health Services. By The Editor .. .. .. .. .. .. .. .. .. | 163 |
| The Twenty-fifth Long Fox Memorial Lecture: Somerset Lake Villages. By Dr. A. Bulleid, F.S.A. .. .. .. .. | 187 |
| Abdominal Pain in Childhood. By Wilfrid Sheldon, M.D., F.R.C.P. | 219 |
| The Treatment of Tobacco Amblyopia by Acetyl-Choline. By B. H. Cragg, L.M.S., M.D. .. .. .. .. .. .. .. | 237 |
| The History of the Bristol Medical Dramatic Club. By A. L. Flemming, M.B., Ch.B. .. .. .. .. .. .. .. | 239 |
| Reviews of Books .. .. .. .. .. .. | 45, 101, 168, 247 |
| Editorial Notes .. .. .. .. .. .. | 54, 117, 175, 257 |
| Obituary .. .. .. .. .. .. .. .. .. | 59 |
| Meetings of Societies .. .. .. .. .. | 61, 118, 180, 260 |
| The Medical Library of the University of Bristol | 62, 119, 181, 263 |
| Publications Received .. .. .. .. .. | 65, 121, 184, 264 |
| Local Medical Notes .. .. .. .. .. | 66, 122, 185, 265 |

### The Medical Library of the University of Bristol,

WITH WHICH IS INCORPORATED

**The Library of the Bristol Medico-Chirurgical Society.**

---

*The following donations have been received since the publication of the list in December,* 1935.

March, 1936.

| | | |
|---|---|---|
| Messrs. Bayer Products Ltd. (1) .. .. | 1 | volume. |
| British Medical Association (2).. .. .. | 1 | ,, |
| Professor R. J. Brocklehurst (3) .. .. | 3 | volumes. |
| Brompton Hospital for Consumption (4) .. | 1 | volume. |
| J. Edmund Clark, Esq. (5) .. .. .. | 3 | volumes. |
| Professor F. H. Edgeworth (6) .. .. | 1 | volume. |
| Dr. J. F. S. Esser (7) .. .. .. .. | 1 | ,, |
| Glasgow Royal Maternity and Women's Hospital (8) .. .. .. .. .. | 1 | ,, |
| Professor E. W. Hey Groves (9) .. .. | 3 | volumes. |
| R. L. St. Harmsworth Memorial Research Fund (10) .. .. .. .. .. .. | 1 | volume. |
| London University (11) .. .. .. .. | 1 | ,, |
| Dr. Iwao Matsuo (12) .. .. .. .. | 1 | ,, |
| Michell Clarke Memorial Fund (13) .. .. | 17 | volumes. |
| Otago University Medical School (14) .. | 1 | volume. |
| W. Roger Williams, Esq., F.R.C.S. (15) .. | 1 | ,, |
| Wilmer Ophthalmological Institute of The Johns Hopkins University (16) .. .. .. | 1 | ,, |

Unbound periodicals have also been received from Professor E. W. Hey Groves, Professor C. Bruce Perry and Dr. J. Odery Symes.

### THE ONE HUNDRED AND FIFTY-FOURTH LIST OF BOOKS.

The figures in round brackets refer to the figures after the names of the donors. The books to which no such figures are attached have either been bought from the Library Fund or received through the *Journal*.

| | | |
|---|---|---|
| **Bray, G. W.** | *Recent Advances in Allergy.* 2nd Ed. (13) | 1934 |
| **Buckley, C. W.** | *Reports on Chronic Rheumatic Diseases* | 1935 |
| *Central Control Board* (*Liquor Traffic*). | *Alcohol: its action on the human organism.* 2 Copies 2nd. Ed. (3) | 1935 |
| **Clark, W. J., and Drinker, P.** | *Industrial Medicine* | 1935 |
| **Clay, H. H.** | *The Sanitary Inspector's Handbook* | 1936 |
| **Cohen, H.** | *New Pathways in Medicine* | 1935 |
| **Craig, C. F.** | *Amebiasis and Amebic Dysentery* (13) | 1934 |
| **Davis, O. C. M., and Wilshire, F. A.** | *Mentality and the Criminal Law* | 1935 |
| **Dodson, A. I.** | *Synopsis of Genito-urinary Diseases* (13) | 1934 |
| **Doxtater, L. W.** | *Procedures in Modern Crown and Bridgework* | 1931 |
| **Duke-Elder, S.** | *Recent Advances in Ophthalmology.* 3rd Ed. (13) | 1934 |
| **Edgeworth, F. H.** | *The Cranial Muscles of Vertebrates* (6) | 1935 |
| **Esser, J. S. F.** | *Biological Flaps* (7) | — |
| **Fishberg, A. M.** | *Hypertension and Nephritis* 3rd Ed. (13) | 1934 |
| **Frahm, F. W.** | *Principles and Technics of Full Denture Construction* | 1934 |
| **Fraser, F. R.** | *Principles of Therapeutics* (13) | 1934 |
| **Fry, H. K.** | *Introduction to General Therapeutics* | 1935 |
| **Gillett, H. W., and Irving, A. J.** | *Gold Inlays by the Indirect System* | 1932 |
| **Griffiths, H. E.** | *Injury and Incapacity* (13) | 1935 |
| **Harrison, T. R.** | *Failure of the Circulation* (13) | 1935 |
| **Haultain, W. F. T., and Kennedy, C.** | *A Practical Handbook of Midwifery and Gynæcology* 2nd Ed. | 1935 |
| **Heffter, A.** | *Handbuch der experimentellen Pharmakologie.* Bd. III. Teil 4. | — |
| **Hindley-Smith, J. D.** | *Chronic streptococcal toxæmia and rheumatism* | 1935 |
| **Hutchison, R.** | *Lectures on Diseases of Children* 7th Ed. | 1936 |
| **Hutchison, R., ed.** | *An Index of Treatment* 11th Ed. (9) | 1936 |
| **Ivy, R. H., and Curtis, L.** | *Fractures of the Jaws* (13) | 1931 |
| **Kennedy, E.** | *Partial Denture Construction* | 1932 |
| **Kretschmer, E.** | *A Textbook of Medical Psychology.* Translated by E. B. Strauss (13) | 1934 |
| **Logan, J. D., and Logan, W. R.** | *Dental Prosthetics* 2nd Ed. | 1934 |
| **Love, R. J. M.** | *Guide to the Surgical Paper, with questions and answers* | 1935 |
| **Macdonald, G., and Hargrave Wilson, W.** | *The Osteopathic Lesion* | 1935 |
| **Maingot, R.** | *Post-graduate Surgery.* Vol. I. (9) | 1936 |
| **Martin, C. R. A.** | *Slums and Slummers* | 1935 |

**Matsuo, I.** .. .. .. *Biologische Untersuchungen über Farbstoffe.* Bd. II. (12) —
**Meaker, R.** .. .. *Human Sterility* .. .. .. .. .. .. (13) 1934
*Medical Practitioners' Handbook (B.M.A.)* .. .. .. .. .. .. .. (2) —
*Medicine in its Chemical Aspects. Reports from the Medico Chemical Research Laboratories of the I. G. Farbenindustrie Aktiengesellschaft.* Vol. II. .. .. .. .. .. .. .. .. .. .. .. .. (1) 1934
**Merritt, A. H.** .. .. *Periodontal Diseases* .. .. .. .. .. .. (14) 1935
**Penrose, L. S.** .. .. *Mental Defect* .. .. .. .. .. .. .. .. .. 1933
**Perry, C. B.** .. .. *Bacterial Endocarditis* .. .. .. .. .. (10) 1936
**Rocha, O. R.** .. .. *Anatomia Quirurgica* .. .. .. .. .. (9) 1935
**Roche, A. E.** .. .. *Medical and other Verses* .. .. .. .. .. 1935
*Rôle of Metals in Intracellular Reactions* .. .. .. .. .. .. (3) 1934
**Roxburgh, A. C.** .. *Common Skin Diseases* .. .. .. 3rd. Ed. 1936
**Savill, A. F.** .. .. *The Hair and the Scalp* .. .. .. .. .. [1935]
**Seldin, H. M.** .. .. *Practical Anesthesia for Dental and Oral Surgery* .. .. .. .. .. .. .. .. .. .. (13) 1934
**Short, A. R., and Ham, C. I.** *A Synopsis of Physiology.* [2 copies] 2nd Ed. 1936
**Sturge, R. F.** .. .. *The Weather at Clifton.* 3 Vols. 1860–1889; 1890–1900; 1901–1911 .. .. .. .. .. —
**Thoma, K. H.** .. .. *Clinical Pathology of the Jaws* .. .. (13) 1934
**Turner, C. R., and Anthony, L. P.** *American Textbook of Prosthetic Dentistry* .. .. .. 6th Ed. (13) 1932
**Watson, F.** .. .. *The Life of Sir Robert Jones* .. .. .. .. 1934
**Weber, F. P.** .. .. *Endocrine Tumours and other Essays* .. .. 1936
**Widdowson, T. W.** .. *Notes on Dental Surgery and Pathology* .. (13) 1921
**Williams, W. R.** .. *Teratoid Tumours* .. .. .. .. .. .. .. (15) —

### TRANSACTIONS, REPORTS, JOURNALS, ETC.

Brompton Hospital Reports. Vol. IV. .. .. .. .. .. .. .. .. (4) 1935
Collected Reprints from the Wilmer Ophthalmological Institute. IV. (16) 1933–35
Medical Report of the Glasgow Royal Maternity and Women's Hospital Medical Report for the year 1933 .. .. .. .. .. .. .. .. (8) 1934
Medical Research Council Special Report, Series 206. (Wilson: Bacteriological Grading of Milk) .. .. .. .. .. .. .. .. .. .. —
Proceedings of the University of Otago Medical School. No. 13. (14) 1936
Report of the Conference of Representatives nominated by the Universities of Oxford, Cambridge and London, the Royal College of Physicians of London, the Royal College of Surgeons of England and the Society of Apothecaries of London, on the Medical Curriculum .. .. .. .. .. .. .. .. .. .. .. .. (11) —
St. Thomas's Hospital Reports. LVII. .. .. .. .. .. .. .. .. .. 1933
Surgical Clinics of North America. XV. 6. .. .. .. .. .. .. .. —
Transactions of the Association of American Physicians. 50 .. .. —
Transactions of the College of Physicians. 4th Series. Vols. I—II —
Transactions of the Ophthalmological Society. LV. .. .. .. .. .. 1935

### Publications Received

From Edward Arnold & Co. :

*Lectures on Diseases of Children.* By Robert Hutchison, M.D., LL.D., F.R.C.P. 7th Edition. Price 21s.

*The Study of Anatomy.* By S. E. Whitnall, M.A., M.D., B.Ch., M.R.C.S., L.R.C.P., F.R.S. (Canada). 3rd Edition. Price 4s. 6d.

From Baillière, Tindall and Cox :

*Surgical Emergencies in Children.* By H. C. Edwards, M.S., F.R.C.S. Price 12s. 6d.

From Hodder & Stoughton Ltd. :

*New Pathways in Medicine.* By Henry Cohen, M.D., F.R.C.P. 1935. Price 1s.

From Hutchinson & Co. :

*Insulin, its Production, Purification, and Physiological Action.* By D. W. Hill, B.Sc., Ph.D., and F. O. Howitt, M.Sc., Ph.D., F.I.C. Price 12s. 6d.

From H. K. Lewis & Co. :

*The Sanitary Inspector's Handbook.* By H. H. Clay, F.R.San.I., F.I.S.E. 2nd Edition. Price 15s.

*Endocrine Tumours and other Essays.* By Frederick Parkes Weber, M.A., M.D., F.R.C.P., F.S.A. Price 7s. 6d.

*Infra-Red Irradiation.* By W. Beaumont, M.R.C.S., L.R.C.P. Price 6s. 6d.

*The Treatment of Asthma.* By F. T. Harrington, M.R.C.S., L.R.C.P. Price 6s.

From Oxford University Press :

*The Tuberculin Handbook.* By Halliday Sutherland, M.D. 1936. Price 7s. 6d.

*Painful and Dangerous Diseases of the Ear.* By R. R. Woods, M.B., F.R.C.S.I. 1936. Price 15s.

*The Natural History of Disease.* By John A. Ryle, M.A., M.D., F.R.C.P. Price 15s.

*Early Diagnosis of Malignant Disease.* By M. Donaldson, F.R.C.S., M.B., B.Ch., F.C.O.G., and others. Price 8s. 6d.

*A Textbook of Psychiatry.* By D. K. Henderson, M.D., F.R.F.P.S., F.R.C.P.E., and R. D. Gillespie, M.D., F.R.C.P., D.P.M. 4th Edition. Price 18s.

From John Wright & Sons Ltd. :

*Synopsis of Physiology.* By A. Rendle Short, B.Sc., M.D., F.R.C.S., and C. I. Ham, M.B., B.Ch., F.R.C.S. 2nd Edition. Price 10s. 6d.

*British Journal of Surgery.* Vol. XXIII. 91. Jan., 1936. Price 12s. 6d.

*Emergency Surgery.* By Hamilton Bailey, F.R.C.S. 2nd Edition. Price 50s.

*An Index of Treatment.* Edited by Robert Hutchison. 11th Edition. Price 42s.

*Bacterial Endocarditis.* By C. Bruce Perry, M.D., M.R.C.P. Price 10s. 6d.

*Diseases of the Nose, Throat and Ear.* Edited by A. Logan Turner, M.D., LL.D., F.R.C.S. Price 20s.

### Local Medical Notes

**The Long Fox Memorial Lecture.**—The twenty-fifth Long Fox Memorial Lecture will be delivered at 8.30 p.m. on Tuesday, 20th October, in the University, by Dr. A. Bulleid, F.S.A. Subject: "Somerset Lake Villages." Admission is free and no tickets are required: but it will be a great convenience if those intending to be present will notify the Secretary, Long Fox Memorial Appointing Board, The University, Bristol.

### ℞ GALENICALS ℞

THE STUDENT SOCIETY OF THE BRISTOL SCHOOL OF MEDICINE.

A new Medical Students' Society has just come into being and has been received with enthusiasm. Its objects are stated to be "to further and co-ordinate the scientific and social interests of the students of the University of Bristol Medical School and to bring about a closer co-operation between the students of the Bristol Royal Infirmary, the Bristol General Hospital, and the Dissecting Room." Ordinary membership is confined to unqualified students, associate membership to past students. The subscribing membership is already over a hundred. Three meetings were held during the Spring Term and the following papers presented:—

14th February. "The South African Native and his Medical Lore," by Professor T. B. Davie.

27th February. "Euthanasia." A debate opened by Dr. C. Corfield.

13th March. "Cheiro-practitioners," by Professor S. E. Whitnall.

All qualified members of the profession are welcome at all meetings, notices of which are posted in advance at the main medical institutions: we hope also to publish the programmes in the *Journal*. The Secretary is Mr. L. Willoughby, 16 Elton Road, Bristol 8.

### Bristol Medico-Chirurgical Society.

### President.

A. E. ILES, *O.B.E.*, M.B., B.S., F.R.C.S.

### President-Elect.

R. C. CLARKE, *O.B.E.*, M.B., Ch.B. Brist.

### Committee.

*Ex-Officio* J. A. NIXON, *C.M.G.*, B.A., M.D. Cantab., F.R.C.P. (Editor of *Journal*).

G. PARKER, M.A., M.D. Cantab. (Hon. Librarian).

*Elected.*

H. ELWIN HARRIS, B.A., M.B. Cantab., F.R.C.S. (Ex-President).

DUNCAN WOOD, F.R.C.S.

A. L. FLEMMING, M.B., Ch.B. Brist.

CLIFFORD A. MOORE, M.B., M.S. Lond.

H. L. FULLER.

P. S. MARTIN, *M.C.*

W. V. WOOD, *M.C.*, B.A. Cantab.

A. FELLS, M.B., C.M. Edin.

R. C. CLARKE, *O.B.E.*, M.B., Ch.B. Brist.

A. L. TAYLOR, M.D. Lond.

### Honorary Secretary.

R. V. COOKE, Ch.M.

### Honorary Treasurer.

A. E. ILES, *O.B.E.*, M.B., B.S. Lond., F.R.C.S.

### University Library Sub-Committee.

G. PARKER, M.A., M.D. Cantab.
A. R. SHORT, B.Sc., M.D. Lond., F.R.C.S.
J. O. SYMES, M.D. Lond.

Medical Practitioners wishing to join the Society are requested to communicate with the Hon. Secretary, Mr. R. V. Cooke, Failand Lodge, Guthrie Road, Clifton. The Annual Subscription is £1 1s., payable to Hon. Treasurer.

---

*Extracts from Journal By-laws.*

4.—The *Journal* shall be delivered free to Subscribers and also to Members of the Society whose subscriptions are not in arrear.

6.—The Annual Subscription to the *Journal* is 10/6, payable to Hon. Treasurer.

---

Communications intended for insertion in the *Journal* should be sent to the Editor, Dr. J. A. Nixon, 7 Lansdown Place, Clifton, Bristol.

Books for Review and Exchange Journals should be sent to the Assistant-Editor, *Bristol Medico-Chirurgical Journal*, Medical Library, The University, Bristol.

Communications referring to the delivery of the *Journal* should be sent to the Editorial Secretary, A. L. Flemming, 48 Pembroke Road, Clifton, Bristol.

Cases for Binding Vols. I—LII are now ready, and may be obtained price 1/9 each (postage extra), upon application to J. W. Arrowsmith Ltd., Quay Street, Bristol; or the *Journal* will be bound, including Case, for 7/- per volume. The inclusive index for Volumes I—L may be obtained, price 3/- (postage extra).

### Bristol Medico-Chirurgical Society.

### PAST PRESIDENTS.

| | |
|---|---|
| 1874—75. | FREDERICK BRITTAN, M.D., B.S. Dub. |
| 1875—76. | ROBERT WILLIAM COE. |
| 1876—77. | SAMUEL MARTYN, M.D. Ed. |
| 1877—78. | AUGUSTIN PRICHARD, M.D. Berlin. |
| 1878—79. | HENRY EDWARD FRIPP, M.D. Würzburg. |
| 1879—80. | WILLIAM MICHELL CLARKE. |
| 1880—81. | JOSEPH GRIFFITHS SWAYNE, M.D. Lond. |
| 1881—82. | EDWARD LONG FOX, M.D. Oxon. |
| 1882—83. | JAMES GEORGE DAVEY, M.D. St. And. |
| 1883—84. | WILLIAM JOHNSTONE FYFFE, M.D., B.A. Dub. |
| 1884—85. | GEORGE FORSTER BURDER, M.D. Aberd. |
| 1885—86. | WILLIAM HENRY SPENCER, M.D., M.A. Cantab. |
| 1886—87. | FRANCIS POOLE LANSDOWN. |
| 1887—88. | LEMUEL MATTHEWS GRIFFITHS. |
| 1888—89. | ROBERT SHINGLETON SMITH, M.D., B.Sc. Lond. |
| 1889—90. | NELSON CONGREVE DOBSON, Ch.M. Brist. |
| 1890—91. | SAMUEL HENRY SWAYNE. |
| 1891—92. | FRANCIS RICHARDSON CROSS, M.B. Lond., LL.D. Brist. |
| 1892—93. | EDWARD MARKHAM SKERRITT, M.D., B.S., B.A. Lond. |
| 1893—94. | JAMES GREIG SMITH, M.B., C.M., M.A. Aberd., F.R.S.E. |
| 1894—95. | ALFRED JAMES HARRISON, M.B. Lond. |
| 1895—96. | ARTHUR WILLIAM PRICHARD. |
| 1896—97. | ALFRED EDWARD AUST LAWRENCE, M.D., C.M. Aberd. |
| 1897—98. | JOHN EDWARD SHAW, M.B., C.M. Ed. |
| 1898—99. | ROBERT ROXBURGH, M.B. Ed. |
| 1899—00. | WILLIAM HENRY HARSANT. |
| 1900—01. | DAVID SAMUEL DAVIES, M.D. Lond. |
| 1901—02. | BARCLAY JOSIAH BARON, M.B., C.M. Ed. |
| 1902—03. | GEORGE MUNRO SMITH, M.D. Brist. |
| 1903—04. | JAMES PAUL BUSH, C.M.G., C.B.E., Ch.M. Brist. |
| 1904—05. | REGINALD EAGER, M.D. Lond. |
| 1905—06. | JOHN DACRE. |
| 1906—07. | JAMES TAYLOR. |
| 1907—08. | HENRY WALDO, M.D., C.M. Aberd. |
| 1908—09. | JOHN MICHELL CLARKE, M.D., M.A. Cantab. |
| 1909—10. | JAMES SWAIN, C.B., C.B.E., M.D., M.S. Lond. |
| 1910—11. | BERTRAM MITFORD HERON ROGERS, M.D., B.Ch., B.A. Oxon. |
| 1911—12. | CHARLES ALEXANDER MORTON. |
| 1912—13. | WALTER CARLESS SWAYNE, M.D., B.S. Lond. and Brist. |
| 1913—14. | PATRICK WATSON-WILLIAMS, M.D. Lond. |
| 1914—15. | WILLIAM HARRY CHRISTOPHER NEWNHAM, M.A., M.B. Cantab. |
| 1915—16. | GEORGE PARKER, M.A., M.D. Cantab. |
| 1916—17. | FRANCIS HENRY EDGEWORTH, M.A., M.D. Cantab., D.Sc. Lond. |
| 1917—18. | FREDERICK LACE. |
| 1918—19. | ROBERT GUTHRIE POOLE LANSDOWN, M.D., B.S. Durh. |
| 1919—20. | I. WALKER HALL, M.D., Ch.B. Vict. |
| 1920—21. | LEOPOLD ERNEST VALENTINE EVERY-CLAYTON, M.D. Lond. |
| 1921—22. | CYRIL HUTCHINSON WALKER, B.A., M.B. Cantab. |
| 1922—23. | JOHN ALEXANDER NIXON, C.M.G., B.A., M.D. Cantab. |
| 1923—24. | JOHN LACY FIRTH, M.D., M.S. Lond. |
| 1924—25. | JOHN ODERY SYMES, M.D. Lond. |
| 1925—26. | THOMAS CARWARDINE, M.S. Lond. |
| 1926—27. | ARTHUR LAUNCELOT FLEMMING, M.B., Ch.B. Brist. |
| 1927—28. | WALTER KENNETH WILLS, M.A., M.B., B.Ch. Cantab. |
| 1928—29. | HENRY LAWRENCE ORMEROD, M.D., B.Ch. R.U.I. |
| 1929—30. | CHARLES FERRIER WALTERS. |
| 1930—31. | DAVID CHARLES RAYNER, Ch.M. Brist. |
| 1931—32. | JOHN ROGER CHARLES, M.A., M.D. Cantab. |
| 1932—33. | ERNEST WILLIAM HEY GROVES, M.D., M.S., D.Sc., B.Sc. Lond. |
| 1933—34. | HERBERT ELWIN HARRIS, B.A., M.B. Cantab. |
| 1934—35. | A. RENDLE SHORT, B.Sc., M.D. Lond., F.R.C.S. |

1936.

### LIST OF SUBSCRIBERS

TO THE

### Bristol Medico-Chirurgical Journal.

### HONORARY MEMBERS OF THE SOCIETY.

| | |
|---|---|
| PARKER, GEORGE, M.A., M.D. Cantab. .. .. .. .. .. .. | 9, Pembroke Road, Clifton, Bristol 8. |
| SWAIN, JAMES, *C.B.*, *C.B.E.*, M.D., M.S. Lond. .. .. .. | Wyndham House, Easton-in-Gordano. |
| WATSON-WILLIAMS, P., M.D. Lond. | 2 Rodney Place, Clifton, Bristol 8. |

### SUBSCRIBERS.

*Those marked * are Members of the Society.*

| | |
|---|---|
| *ACKLAND, W. R., M.D.S. .. .. .. .. .. .. | 5 Rodney Place, Clifton, Bristol 8. |
| *ADAMS, A. W., M.S. Lond. .. .. .. .. .. | Rodney House, Clifton, Bristol 8. |
| ADAMS, J. B. .. .. .. .. .. .. .. .. .. | Kent Lodge, Eastbourne. |
| *ADAMS, S.B., Ph.D., M.B., Ch.B. Brist. .. .. | 19 Charlotte Street, Bristol 1. |
| *ALDWINCKLE, H. M., M.B., Ch.B. Brist. .. | 1 Manor Road, Fishponds, Bristol. |
| *ALEXANDER, A. J. P., M.D. Belf. .. .. .. .. | Winsley Sanatorium, near Bath. |
| *ALEXANDER, D. A., M.B., Ch.B. Vict. .. .. | 112 Pembroke Road, Clifton, Bristol 8. |
| ALEXANDER, G. L., B.A., M.B., B.Ch. Cantab. | West African Medical Service, Laura, via Accra, Gold Coast. |
| *ALFORD, A. F., M.B., Ch.B. Brist. .. .. .. | Central Hotel, Bristol 1. |
| ALFORD, H. J., M.D. Lond. .. .. .. .. .. | 4 Park Terrace, Taunton. |
| *ARMSTRONG, JEAN, M.B., Ch.B. Brist. .. .. | Succoth, Duckmoor Road, Ashton Gate, Bristol 3. |
| ASHTON, L. P., M.B., Ch.B. Brist. .. .. .. | Kapsowar, P.O. Eldoret, Kenya, East Africa. |
| *AUBREY, T., M.B. Lond. .. .. .. .. .. .. | Bitton, near Bristol. |
| *BAIN, W. .. .. .. .. .. .. .. .. .. .. | 1 Greville Road, Southville, Bristol 3. |
| *BAKER, LILY A., M.D., B.Ch., B.A.O., R.U.I. | Clifton Down House, Beaufort Buildings, Clifton, Bristol 8. |
| *BARBER, M. C., M.B., Ch.B. Brist. .. .. .. | Ingledene, High Street, Staple Hill, Bristol. |
| *BARKER, G. H., M.D., B.Sc. Lond. .. .. .. | 124 Redland Road, Bristol 6. |
| *BARTHOLOMEW, E. U. .. .. .. .. .. .. .. | 12 Lansdown Place, Clifton, Bristol 8. |
| *BATES, R. M. .. .. .. .. .. .. .. .. .. | Purdown House, Stapleton, Bristol 5. |
| BEATSON, D. H., M.B., Ch.B. Brist. .. .. | 8 Richmond Park Road, Clifton, Bristol 8. |
| *BERGIN, F. G. .. .. .. .. .. .. .. .. | 31 Oakfield Road, Clifton, Bristol 8. |
| *BERRY, R. J. A., Prof., M.D., C.M. .. .. | Director of Medical Services, Stoke Park Colony, Bristol 5. |
| *BIRRELL, J. A., M.D., M.S. Lond. .. .. .. | 36 Kellaway Avenue, Bristol 6. |
| *BLACHFORD, J. V., *C.B.E.*, M.D. Durh. .. .. | Milverton House, Long Ashton, near Bristol. |
| BLACKETT, J. F., M.D., B.S. Lond. .. .. .. | Hamsterley, Bloomfield Park, Bath. |
| BLETCHLY, G. PLAYNE, M.B. Lond. .. .. | c/o Lloyds Bank, Nailsworth, Glos. |
| *BODMAN, A. P., M.B., Ch.B. .. .. .. .. | 11 Hurle Crescent, Clifton, Bristol 8. |
| *BODMAN, C. O., M.D. Durh. .. .. .. .. | 17 Upper Belgrave Road, Clifton, Bristol 8. |
| *BODMAN, F. H., M.B., Ch.B. Brist. .. .. .. | 2 Redland Court Road, Bristol 6. |
| *BODMAN, J. HERVEY, M.D. Lond. .. .. .. | 11 Hurle Crescent, Clifton, Bristol 8. |
| BOLT, R. F. .. .. .. .. .. .. .. .. .. | 70 Great Russell Street, London, W.C.1. |
| *BOULTON, B. J., M.B., Ch.B. Brist. .. .. | Ham Green Hospital, Pill, near Bristol. |
| *BOWN, N. J., M.B., Ch.B. Brist. .. .. .. | Demeter, Wraxall, Somerset. |
| *BRADBEER, E. G., M.B., Ch.B. Brist. .. .. | 13 Percival Road, Bristol 8. |
| *BRADLEY, W. H., B.A., D.M. Oxon. .. .. | St. Wulstan's, Stratton-on-Fosse, Bath. |
| BRASHER, C. W. J. .. .. .. .. .. .. .. | Woodlands Park, Great Missenden, Bucks. |

| | |
|---|---|
| *BRINCKMAN, W. P., M.B., Ch.B. Brist. | 4 Oakland Road, Redland, Bristol 6. |
| *BRITTON, R. B., M.B., B.S. Lond. | 33a Whiteladies Road, Clifton Bristol 8. |
| *BROCKLEHURST, R. J., M.A., D.M. Oxon. | The University, Bristol 8. |
| BROWN, J. E., M.B., Ch.B. Brist. | 137 Belmont Circular Road, Port of Spain, Trinidad, B.W.I. |
| *BUCKMASTER, G. A., M.A., M.D. Oxon. | 6 Victoria Square, Clifton, Bristol 8. |
| *BURGES, R. | Druid's Mead, Stoke Bishop, Bristol 9. |
| *BURGESS, N. F. C., M.D. Cantab. | Hereford House, Clifton, Bristol 8. |
| *BURNETT, R. H., M.B., B.S. Durh. | 528 Bath Road, Brislington, Bristol 4. |
| *BUSH, G. B., M.B., Ch.B. Brist. | 132 Queen's Road, Clifton, Bristol 8. |
| | |
| *CAIRNS, F. J. | Devon House, Whitehall Road, Bristol 5. |
| *CAREY, R. S., *O.B.E.*, B.A. Cantab. | 502 Bath Road, Brislington, Bristol 4. |
| *CARLETON, H. H., M.A., M.D., B.Ch. Oxon. | 1 Lansdown Road, Clifton, Bristol 8. |
| *CASSON, E., M.D., Ch.B. Brist. | Dorset House, Clifton Down, Bristol 8. |
| *CATES, H. J., M.D. Lond. | Northwoods House, Winterbourne, Bristol. |
| *CHAMBERS, E. R. | 9 Clifton Park, Clifton, Bristol 8. |
| *CHARLES, J. R., M.A., M.D. Cantab. | 5 Rodney Place, Clifton, Bristol 8. |
| *CHESTERMAN, J. T., M.D., B.S. | Royal Hospital Sheffield. |
| *CHITTY, H., M.S. Lond. | 46 Pembroke Road, Clifton, Bristol 8. |
| *CHRISTOFFERSON, P. E., M.B., Ch.B. Brist. | 4 Victoria Square, Clifton, Bristol 8. |
| *CLAREMONT, L. E., M.D.S. Brist. | 5 Rodney Place, Clifton, Bristol 8. |
| *CLARKE, R. C., *O.B.E.*, M.B., Ch.B. Brist. | 29 Victoria Square, Clifton, Bristol 8. |
| CONNELLAN, P. S. | Marmion, Shortheath Road, Farnham, Surrey. |
| *COOKE, ELIZABETH M., M.D. | Failand Lodge, Guthrie Road, Clifton. |
| *COOKE, R. V., Ch.M. | Failand Lodge, Guthrie Road, Clifton. |
| *COOKSON, R. G. | 5 Worcester Road, Clifton, Bristol 8. |
| *COOMBS, N. C. | 14 Southfield Road, Westbury-on-Trym, Bristol. |
| *COOPER, WILLIAM RUSSELL | 13 Westfield Park, Redland, Bristol 6. |
| *CORFIELD, C., M.D. Durh. | 5 Kensington Place, Clifton, Bristol 8. |
| *CORNER, BERYL, M.B., B.S. | The Children's Hospital, Bristol. |
| *COSSHAM, W. L., M.B., Ch.B. Brist. | 3 Claremont Road, Bishopston, Bristol 7. |
| *COURTNEY, R. M., M.B., Ch.B. Glasg. | 487 Gloucester Road, Bristol 7. |
| *COXON, BEATRICE | 16 Richmond Hill, Clifton, Bristol 8. |
| *CRAIG, A. A. (Miss), M.B., B.S. | 18 Vyvyan Terrace, Clifton, Bristol 8. |
| *CROOK, B. A., M.B., Ch.B. Brist. | Timsbury. |
| *CROSSMAN, F. W., M.B. Durh. | White's Hill, Hambrook, near Bristol. |
| *CROSS, CLARA D., M.D. | 17 Midford Road, Odd Down, Bath. |
| | |
| *DATTA, S. S., M.D. Brist. | Snowdon House, Fishponds, Bristol. |
| *DAVIE, T. B., M.D. Liverpool | Canynge Hall, Clifton, 8. |
| *DAVIES, E. D. D. | Standish House, Stonehouse, Glos |
| *DAWSON, W. R., *O.B.E.*, M.D. Dub. | 18 Brock Street, Bath. |
| *DELANEY, N., M.B., Ch.B. | Charlesworth, Bishopsworth, Bristol. |
| DEVINE, H., *O.B.E.*, M.D. Lond. | Holloway Sanatorium, Virginia Water. |
| *DICK, A., M.B., Ch.B. Glasg. | 14 Owen Grove, Henleaze, Bristol. |
| *DICKSON, W. A., M.D. | Standish House, Stonehouse, Glos. |
| *DIX, C. | 11 Victoria Square, Clifton, Bristol 8. |
| *DIXON, HELEN M., M.B., Ch.B. Brist. | 10 Whatley Road, Clifton, Bristol 8. |
| *DIXON, T. B. | 11 Pembroke Road, Clifton, Bristol 8. |
| *DREW, J. H., M.D. | Penlee, Uphill Road, Weston-super-Mare. |
| *DUERDEN, J. R., M.B., Ch.B. Brist. | 149 Bell Hill Road, St. George, Bristol 5. |
| *DUTTON, HILDA R. | 405 Stapleton Road, Bristol 5. |
| *DYER, WALTER PERCY | West View, Westbury-on-Trym, Bristol. |
| | |
| *EGLINTON, J. H. C. | Street, Somerset. |
| ELLIOT BROS. LTD. | 20–22 O'Connell Street, Sydney, N.S.W., Australia. |
| ELLIOTT, F. PERCY, M.B., C.M. Aberd. | 113 Grove Road, Walthamstow London, E. 17. |

| | |
|---|---|
| *ELLIOTT, W. H. A., M.B., B.S. Lond. .. .. | 116 Pembroke Road, Clifton, Bristol 8. |
| *EYRE-BROOK, A. L., M.B., B.S. (Honrs.) Lond. | The Cottage, Portishead. |
| *EVANS, G. M., M.B., Ch.B. .. .. .. .. | 117 Ashley Road, Bristol. |
| *FALLON, Col. J. .. .. .. .. .. .. .. .. | 35 Henleaze Gardens, Henleaze, Bristol. |
| *FARAKER, E. C. .. .. .. .. .. .. .. .. | 85 Coldharbour Road, Westbury Park, Bristol 6. |
| *FARQUHARSON, J. L. .. .. .. .. .. .. .. | Breda, Weston-super-Mare. |
| *FAWN, G. F. .. .. .. .. .. .. .. .. .. | 6 Oakfield Road, Clifton, Bristol 8. |
| *FELLS, A., M.B., C.M. Edin. .. .. .. .. | 6 Elton Road, Clifton, Bristol 8. |
| *FELLS, G. R., M.B., Ch.B. Brist. .. .. .. | 19 Southmead Road, Bristol. |
| *FELLS, R. R., M.B., B.S. Lond. .. .. .. | 17 Mortimer Road, Clifton, Bristol 8. |
| *FENELEY, G. L., M.B., Ch.B. .. .. .. .. | Englewood, Falcondale Road, Westbury-on-Trym. |
| *FINZEL, H. F. M., M.D. Brist. .. .. .. .. | The Beeches, St. Anne's Park, Bristol. |
| *FIRTH, J. L., M.D., M.S. Lond. .. .. .. | 8 Victoria Square, Clifton, Bristol 8. |
| *FISHER, A. C., M.B., Ch.B. Brist. .. .. .. | Roan Antelope Mine, N. Rhodesia. |
| *FITZGIBBON, G. M., M.D. .. .. .. .. .. | 52 Pembroke Road, Clifton, Bristol 8. |
| *FITZGIBBON, Mrs. L. P., M.B., Ch.B. .. .. | 52 Pembroke Road, Clifton, Bristol 8. |
| *FLEMMING, A. A. G. .. .. .. .. .. .. .. | Manver's House, Bradford-on-Avon. |
| *FLEMMING, A. L., M.B., Ch.B. Brist. .. .. | 48 Pembroke Road, Clifton, Bristol 8. |
| *FLEMMING, C. E. S. .. .. .. .. .. .. .. | Manver's House, Bradford-on-Avon. |
| *FORRESTER-BROWN, MAUD, M.D., M.S. Lond. | 22 Coombe Park, Bath. |
| *FOSS, G. L., M.B., B.Ch. Camb. .. .. .. | Cloud's Hill House, St. George, Bristol 5. |
| *FOX, F. E., B.A. Camb. .. .. .. .. .. .. | Brislington House, Brislington, Bristol 4. |
| *FRASER, A. D., M.A., M.B., Ch.B. Glasg. .. | 4 Alexandra Road, Clifton, Bristol 8. |
| *FRASER, D., M.B., Ch.B. Edin. .. .. .. | Westgate, Dursley, Glos. |
| *FULLER, H. L. .. .. .. .. .. .. .. .. | 9 Gay Street, Bath. |
| *FURNIVAL, Col. C. H. .. .. .. .. .. .. .. | "Yatton," Wellington Hill, Horfield, Bristol 7. |
| *GARDEN, R. R., M.A., M.B., B.Ch. Aberd. .. | 9 Harley Place, Clifton, Bristol 8. |
| GARLAND, E. B., M.B., C.M. Edin. .. .. .. | 114a Hampton Road, Redland, Bristol 6. |
| *GEE, C. A. H., M.B. Ch.B. Brist. .. .. .. | Wansdyke, Portbury, near Bristol. |
| GIBBS, J. R. .. .. .. .. .. .. .. .. .. | 48 Codrington Road, Bishopston, Bristol 7. |
| *GLENNY, E. T., M.B., B.S. Lond. .. .. .. | 102 Pembroke Road, Clifton, Bristol 8. |
| *GORDON, R. G., M.D., B.Sc. Edin. .. .. .. | 9 The Circus, Bath. |
| *GORHAM, A. P., M.B., Ch.B. Brist. .. .. .. | 53 Westbury Road, Westbury-on-Trym. |
| *GORNALL, W. A., M.D. Brist. .. .. .. .. | Orchard House, Nailsea, Somerset. |
| *GRAY, J. D. A., M.B., Ch.B. Edin., B.Sc. .. | 212 Redland Road, Bristol 6. |
| *GREEN, S. B., M.B. Lond. .. .. .. .. .. | The Black Wicket, Westbury-on-Trym. |
| *GREENLAW, MADGE E., M.B., Ch.B. Brist. .. | 194 Redland Road, Bristol 6. |
| GRIFFITHS, CORNELIUS A. .. .. .. .. .. | 35 Newport Road, Cardiff. |
| *GROVES, E. W. HEY, M.D., M.S. Lond., D.Sc. .. | 25 Victoria Square, Clifton, Bristol 8. |
| *GUIRDHAM, A., M.A., B.M., B.Ch. Oxon. .. | Bailbrooke House, Bath. |
| *HALL, E. GEORGE, M.B. Lond. .. .. .. .. | 42 Apsley Road, Clifton, Bristol 8. |
| *HAM, C. I. M.B., Ch.B. Brist. .. .. .. .. | Murray Downs, Rhos Road, Rhos-on-Sea North Wales. |
| *HARRIS, H. ELWIN, M.A., M.B. Cantab. .. | The Mount, Halse, Taunton. |
| *HARRIS, H. E., M.A., M.B., B.Ch. Cantab. .. | 13 Lansdown Place, Clifton, Bristol 8. |
| HARRISON, C. C. .. .. .. .. .. .. .. .. | 44 High Street, Keynsham. |
| *HARTLEY, G., M.B., B.S. Lond. .. .. .. | 2 Harley Place, Clifton, Bristol 8. |
| *HEALES, J. N. .. .. .. .. .. .. .. .. | Holmcroft, Street, Somerset. |
| *HECTOR, F., M.D. Lond. .. .. .. .. .. | 1 Victoria Square, Clifton, Bristol 8. |
| *HEMPHILL, R., M.B., B.Ch. .. .. .. .. | Fishponds Mental Hospital, Bristol. |
| *HENDERSON, JAMES, M.B., Ch.B. Aberd. .. | The City Sanatorium, Yardley Road, Birmingham. |
| *HERAPATH, C. E. K., *M.C.*, M.D. Lond. .. | Ormlie, Clifton, Bristol 8. |
| *HERON, GORDON, M.B., Ch.B. Brist. .. .. | 34 Gloucester Road, Bristol 7. |
| *HERON, Mrs. GORDON, M.B., Ch.B. Brist. .. | 34 Gloucester Road, Bristol 7. |
| *HILL, WINIFRED, M.B., Ch.B. .. .. .. | 16 Hilltop Road, West Hampstead, N.W.16. |
| *HOOKER, J. A., M.B., Ch.B. Brist. .. .. .. | 16 St. Edyth's Road, Sea Mills 9. |
| *HOSKEN, J. G. F... .. .. .. .. .. .. .. | Uley, Glos. |

HOSPITAL, BRISTOL GENERAL (*Secretary*, T. W. GREGG).
*HOUGHTON, LYDIA M., M.B., B.S. Lond. .. 74 Church Road, Redfield, Bristol 5.
*HOWELL, H. .. .. .. .. .. .. .. .. .. Kincraig, Beach Road, Weston-super-Mare.
*HUGHES, F. W. TERRELL, M.B., Ch.B. Brist. Chew Magna, Somerset.
*HUGHES, MARGUERITE G., M.B., Ch.B. Brist. Tower House, Whiteladies Road, Bristol.
*HUNTER, F. S. .. .. .. .. .. .. .. .. c/o Messrs. J. Wright & Sons Ltd., Publishers, Bristol 1.
*HYATT, ANNIE W., M.B., B.S. Lond. .. .. 2 Waterloo Road, Shepton Mallet, Som.

*ILES, A. E., *O.B.E.*, M.B., B.S. Lond. .. .. 17 Victoria Square, Clifton, Bristol 8.
INFIRMARY, BRISTOL ROYAL (*Secretary*, E. C. SMITH).
INGLE, C. DURBAN .. .. .. .. .. .. .. Somerton, Somerset.

*JACKMAN, W. A., M.B., Ch.B. Brist. .. .. 15 Mortimer Road, Clifton, Bristol 8.
*JAMES, APRIL D., M.B., Ch.B. Brist. .. .. 8 Johnstone Street, Bath.
*JAMES, J. A, M.D. Lond. .. .. .. .. .. .. 5 Rodney Place, Clifton, Bristol 8.
*JAMES, J. A. Senr. .. .. .. .. .. .. .. 43 Nevil Road, Bishopston, Bristol.
*JENKINS, F. G., M.B., Ch.B. Brist. .. .. .. 51 Redcliff Hill, Bristol 1.
*JENKINS, R. D. .. .. .. .. .. .. .. .. The Copper Beeches, Almondsbury, near Bristol.
*JOHNSON, R. G., M.B. Lond. .. .. .. .. 119 Redland Road, Bristol 6.
*JOLL, C. A., M.D., M.S. Lond. .. .. .. .. 64 Harley Street, London, W.1.
*JORDAN, L. R., M.B., Ch.B. Brist. .. .. .. National Temperance Hospital, Hampstead Road, N.W.1.
*JOSCELYNE, P. C., M.B., Ch.B. Brist. .. .. 70 Longmead Avenue, Bishopston, Bristol 7.

*KEMM, N. F., M.B., B.S. Lond. .. .. .. 200 Redland Road, Durdham Park Bristol 6.
KING, E. F. .. .. .. .. .. .. .. .. .. 2 Upper Wimpole Street, London, W.1.
*KINGSTON, S. H., M.B., Ch.B. Brist. .. .. 3 Whiteladies Road, Clifton, Bristol 8.
*KYLE, H. G., M.A., M.D., B.Ch. Oxon. .. .. 31 Westbury Road, Bristol.

*LACE, F .. .. .. .. .. .. .. .. .. 5 Gay Street, Bath.
LALONDE, T. P. .. .. .. .. .. .. .. .. Wykeham House, Romsey, Hants.
*LEES, E. LEONARD, M.D., C.M. Edin. .. .. 22 Richmond Hill, Clifton, Bristol 8.
*LEGAT, R. EDDOWES, M.B., C.M. Edin. .. .. Murray House, Staple Hill, Bristol.
*LEVIS, J. S.. *M.C.*, M.B., B.Ch., N.U.I. .. 20 Gay Street, Bath.
*LEWIS, F. J., M.B., Ch.B. .. .. .. .. The Royal Infirmary, Bristol.
*LINTON, MARION S., M.B. Lond. .. .. .. 1 Brecon Road, Henleaze, Bristol.
*LISTER, A. E. J., M.B., B.S. Lond. .. .. 86 Pembroke Road, Clifton, Bristol 8.
*LISTER, L. MARGARET .. .. .. .. .. .. 12 All Saints Road, Clifton, Bristol 8.
*LOGAN, H. B. .. .. .. .. .. .. .. .. Elmwood, Aberdeen Road, Cotham, Bristol 6.
*LOXTON, S. D., M.B., Ch.B. .. .. .. .. The Royal Infirmary, Bristol.
*LUCAS, J. E. .. .. .. .. .. .. .. .. .. 48 Coronation Road, Bristol 3.
*LUCAS, J. J. S., M.D. Lond. .. .. .. .. 186 Wells Road, Totterdown, Bristol 4.
*LUCAS, J. V. .. .. .. .. .. .. .. .. .. 186 Wells Road, Knowle, Bristol 4.
*LUCAS, R. P., M.B., Ch.B. Brist. .. .. .. 15 Edgecumbe Road, Redland, Bristol 6.
*LYSAGHT, A. C. .. .. .. .. .. .. .. .. 8 Clifton Hill, Clifton, Bristol 8.

*MCCREA, PHILLIP, M.D., B.Ch. Dub. .. .. 56 Kellaway Avenue, Bristol 6.
*MCKENZIE, W. G., *M.C.* .. .. .. .. .. 10 Woodland Road, Surbiton.
*MACLACHLAN, A. M., M.B., Ch.B. Glasg. .. 65 Redcliff Hill, Bristol 1.
*MCLANNAHAN, J. G. .. .. .. .. .. .. .. The Mount, Stonehouse.
MACLEAN, SIR EWEN J., M.D., C.M. Edin. .. 12 Park Place, Cardiff.
*MACLEOD, DONALD, M.B., C.M. Edin. .. .. 84 Redland Road, Redland, Bristol 6.
*MACLEOD, G., M.A. Glas., M.D., M.B. .. .. Wargrave, Clevedon, Somerset.
*MACWATTERS, J. C., *M.B.E.* .. .. .. .. Almondsbury, near Bristol.
*MARSHALL, A. H., M.B., Ch.B. Brist. .. .. Odelos Canford Lane Bristol.
MARSHALL, A. L., M.B., B.A. Cantab. .. .. Laxfield, Woodbridge, Suffolk.
*MARTIN, P. S. .. .. .. .. .. .. .. .. Victoria Quadrant, Weston-super-Mare.
*MAYES, F. J A. .. .. .. .. .. .. .. .. 23 Pembroke Road, Clifton, Bristol 8.
*MEADOWS, G. .. .. .. .. .. .. .. .. W. D. & H. O. Wills, No. 1 Factory, East Street, Bedminster, Bristol 3.
MIALL, C. L'O .. .. .. .. .. .. .. .. Elm Hayes, Paulton, near Bristol.

| | |
|---|---|
| *MILLING, T., M.B., Ch.B. Belf. .. .. .. .. .. | 1 Vicarage Road, Southville, Bristol 3. |
| *MILNER, A., M.B., B.Ch. Dub. .. .. .. .. .. | 61 Cotham Brow, Bristol 6. |
| *MOORE, C. A., M.B., M.S. Lond. .. .. .. | 56 St. Paul's Road, Clifton, Bristol 8. |
| *MOORE, L. A., M.B., Ch.B. .. .. .. .. | Hillsborough, Cotham Park, Bristol 6. |
| *MOORE, R. H., M.B., Ch.B. .. .. .. .. | Kismet, Bedminster Road, Bristol. |
| *MORGANS, C. C., M.B., Ch.B. .. .. .. .. | 17 First Avenue, St. Anne's Park, Bristol 4. |
| *MORRIS, L. N., M.B., Ch.B. Brist. .. .. .. | 24 All Hallows Road, Upper Easton. Bristol 5. |
| *MUNDY, G. S., M.B., B.Ch. Brist. .. .. .. | Homewood, Coombe Dingle, Bristol. |
| *MYLES, G. T... .. .. .. .. .. .. .. .. | 7 Apsley Road, Clifton, Bristol 8. |
| *MYLES, Q. St. L., M.A., B.M., B.Ch. Oxon. .. | 7 Apsley Road, Clifton, Bristol 8. |
| *NEWLANDS, H. J. .. .. .. .. .. .. .. | Moray House, Fishponds, Bristol. |
| *NICHOLSON-LAILEY, J. R. .. .. .. .. .. .. | 8 Mount Terrace, Taunton. |
| *NIXON, DOREEN, M.B., B.S. .. .. .. .. | 7 Lansdown Place, Clifton, Bristol. |
| *NIXON, J. A., *C.M.G.*, M.D. Cantab. .. .. | 7 Lansdown Place, Clifton, Bristol 8. |
| *NOBLE, J. INNES, M.B., Ch.B. Liverp. .. .. | 102 Pembroke Road, Clifton, Bristol 8. |
| *NORMAN, R. M., M.D., D.P.M. .. .. .. | 25 Victoria Square, Clifton, Bristol 8. |
| *NOTT, WINIFRED, M.B., Ch.B. Brist. .. .. | Fassiefern, 8 Rylestone Grove, Stoke Bishop, Bristol 9. |
| *O'DONOHOE, MONICA A., M.B., Ch.B. .. .. | 19 Westfield Park, Redland, Bristol 6. |
| *ORMEROD, G. L. M.A., M.B., B.Ch. Cantab .. | 2 Henleaze Road, Westbury-on-Trym, Bristol. |
| *ORR-EWING, H. J., *M.C.*, M.D. Lond. .. .. | Beaufort Buildings, Clifton, Bristol 8. |
| *PARKES, J. A., M.B., Ch.B. Liverp. .. .. | 8 South Road, Redland, Bristol 6. |
| PARKINSON, Lt.-Col. G. S., *D.S.O.*, R.A.M.C. | R.A.M.C. Mess, Grosvenor Place, London, S.W.1. |
| *PARRY, R. H., M.B., B.S. Lond. .. .. .. | Public Health Offices, 40 Prince Street, Bristol 1. |
| PARSONS, Sir J. H., M.B., B.S., B.Sc. Lond... | 54 Queen Anne Street, London, W. |
| *PAUL, R. G. .. .. .. .. .. .. .. .. .. | 23 Pembroke Road, Bristol 2. |
| *PERRY, BRUCE, M.D., Ch.B. Brist. .. .. .. | 4 Richmond Park Road, Clifton, Bristol 8. |
| *PHILLIPS, P., M.B., Ch.B. Brist. .. .. .. | Southmead Hospital, Bristol. |
| *PINEO, E. D. .. .. .. .. .. .. .. .. | Richmond House, Langford, Som. |
| *POLLARD, J., M.B., B.Ch., B.A.O., N.U.I .. | 88 Coronation Road, Bristol 3. |
| PORTER, C. P. .. .. .. .. .. .. .. .. | 28 Mill Street, Kidderminster. |
| *POTTER, MABEL F., M.B., Ch.B. .. .. .. | 79 Pembroke Road, Clifton, Bristol 8. |
| *POWELL, H. F., M.D. Brux. .. .. .. .. | Ellenborough Park, Weston-super-Mare. |
| *PRICE, C. H. G., M.B., Ch.B. Brist. .. .. | 6 Clifton Down Road, Bristol 8. |
| *PRICE, N. L., M.B., Ch.B. Brist. .. .. .. | 37 Henleaze Avenue, Bristol. |
| *PRIDIE, K. H., M.B., B.S. Lond. .. .. .. | 40 Apsley Road, Clifton, Bristol 8. |
| *PRINGLE, Miss J. L., M.B., Ch.B. Edin. .. | 115 Lower Oldfield Park, Bath. |
| *PROWSE, D. C., M.B., Ch.B. Brist. .. .. .. | Wigmore House, Thornbury, near Bristol. |
| *PYKE, H. D., M.B., Ch.B. Brist. .. .. .. | 88 Redland Road, Bristol 6. |
| *RANKIN, P. C., M.B., Ch.B. Glasg. .. .. .. | Lawrence Hill House, Bristol. |
| *RAYNER, D. C., Ch.M. Brist. .. .. .. .. | 9 Lansdown Place, Clifton, Bristol 8. |
| *REDMAN, ETHEL, M.B., Ch.B. .. .. .. .. | 55 Bridgwater Road, Bedminster Down, Bristol 3. |
| *ROBERTSON, D., M.B., Ch.B. Edin. .. .. .. | 205 Wells Road, Knowle, Bristol 4. |
| *ROBERTSON, L. G., M.B., Ch.B. .. .. .. | 162 Redland Road, Bristol 6. |
| *ROGERS, HERBERT, M.D. Brist. .. .. .. | 91 Old Park Ridings, Grange Park, N.21. |
| *ROLFE, R., B.A., M.B., B.C Cantab. .. .. | Park House, St. Andrew's Road, Avonmouth. |
| *ROWLEY, A. T. .. .. .. .. .. .. .. .. | 13 Walliscote Road, Weston-super-Mare. |
| *RUDOLF, G. R. A. de M. .. .. .. .. .. | Brentry Colony, Westbury-on-Trym, Bristol. |
| *SAXBY, IDA, D.Sc. Lond. .. .. .. .. .. | 3 Westbury Park, Bristol 6. |
| *SCARFF, G., M.B., Ch.B. Edin. .. .. .. .. | 83 Pembroke Road, Clifton, Bristol 8. |
| *SHAW, J. E., M.B., C.M. Edin. .. .. .. | 23 Caledonia Place, Clifton, Bristol 8. |
| *SHEPHERD, H. L., M.B., Ch.M. Brist. .. .. | 79 Pembroke Road, Clifton, Bristol 8. |
| *SHORT, A. R., B.Sc., M.D., B.S. Lond. .. .. | 69 Pembroke Road, Clifton, Bristol 8. |

| | |
|---|---|
| *SMITH, G. COCKBURN, M.D. Brux. .. .. .. | 12 South Road, Newton Abbot. |
| *SMITH, T. WILSON, M.D. Lond. .. .. .. | 26 The Circus, Bath. |
| *SMYTHE, H. J. D., *M.C.*, M.D., M.S. Lond. .. | 15 Eaton Crescent, Clifton, Bristol 8. |
| *SOMERS, MARY A. E. .. .. .. .. .. .. .. | 2 Ashcombe Road, Weston-super-Mare. |
| *SPOOR, A., M.B., B.Ch. Cantab. .. .. .. | The Hydro, Bristol 1. |
| *STANILAND, M. F... .. .. .. .. .. .. .. | 255 Stapleton Road, Bristol 5. |
| *STATHAM, R. S. S., *O.B.E.* M.D., M.Ch. Brist. | Cheddar, Som. |
| *STEVEN, G. D., M.B., Ch.B. Edin., D.P.H. .. | 11 North Parade, Bath. |
| *STONE, D. M., M.D., B.S. Lond. .. .. .. | 2 Harley Place, Clifton, Bristol 8. |
| *STROVER, H. W. M., *O.B.E.*, M.B., Ch.B. Aberd. | 12 Gloucester Road, Bishopston, Bristol 7. |
| *STRUTHERS, A. J., M.B., Ch.B. Glasg. .. .. | 100 Church Road, Redfield, Bristol 5. |
| *SUTTON, G. E. F., M.D. Lond. .. .. .. .. | 1 Pembroke Road, Clifton, Bristol 8. |
| *SYMES, J. O., M.D. Lond. .. .. .. .. .. | 6 Pembroke Vale, Clifton, Bristol 8. |
| *TASKER, D. G. C., M.B., M.S. Lond. .. .. | 9 College Fields, Clifton, Bristol 8. |
| *TAYLOR, A. L., M.D. Lond. .. .. .. .. | The General Hospital, Bristol 1. |
| TAYLOR, A. L. .. .. .. .. .. .. .. .. .. | The Royal Infirmary, Bristol 1. |
| *TAYLOR, CONSTANCE L., M.B., Ch.B. Edin. .. | Queen Charlotte's Hospital, Marylebone Road, N.W.1. |
| *TAYLOR, H. N., M.A. Cantab., M.D. Dub. .. | Hillside, Ebbw Vale. |
| *TEMPLE, G. H., M.B., C.M. Edin. .. .. .. | Ailanthus, Weston-super-Mare. |
| THIN, JAMES .. .. .. .. .. .. .. .. .. .. | 54 and 55 South Bridge, Edinburgh. |
| *THOMAS J. D., M.B. Cantab. .. .. .. .. | Dan-y-Graig, Cheyne Road, Stoke Bishop, Bristol 9. |
| *TODD, A, T., *O.B.E.*, M.B., Ch.B. Brist. .. | Royal Park House, Clifton, Bristol 8. |
| TRIPP, DOROTHY M. H. .. .. .. .. .. .. | Methodist Missionary Hospital, Karin Nazar, H.E.H. Nizam's Dominions, India. |
| *TRIST, J. R. R., *M.C.* .. .. .. .. .. .. | 120 Henleaze Road, Henleaze. |
| *TRYON, VICTORIA S., M.B., Ch.B. Brist. .. | 3 Harley Place, Clifton, Bristol 8. |
| *WAGHORN, J. .. .. .. .. .. .. .. .. | Royal Empire Society, Queen's Road, Clifton, Bristol 8. |
| *WALBANK, A., M.B., Ch.B. Leeds .. .. .. | 30 Whiteladies Road, Clifton, Bristol 8. |
| *WALKER, CYRIL H., B.A., M.B. Cantab. .. | Harewood, Clapton-in-Gordano, Som. |
| *WALTERS, C. F. .. .. .. .. .. .. .. .. | 5 Mortimer Road, Clifton, Bristol 8. |
| *WANSBROUGH, T. B., M.B., Ch.B. Brist. .. | 8 Mortimer Road, Clifton, Bristol 8. |
| WAKE, S. C. .. .. .. .. .. .. .. .. .. | Brantwood, Bideford, N. Devon. |
| *WARD, H. W. .. .. .. .. .. .. .. .. | Chipping Manor, Wootton-under-Edge. |
| *WARREN, R., M.A., M.D. Oxon. .. .. .. | Ellenborough Hall, Weston-super-Mare. |
| *WATERHOUSE, R,, M.D. Lond. .. .. .. .. | 3 The Circus, Bath. |
| *WATSON-WILLIAMS, E., *M.C.*, B.A., M.B., Ch.M. Cantab. | 12 Victoria Square, Clifton, Bristol 8. |
| WATTS, J. B. V. .. .. .. .. .. .. .. .. | 6 Queen Street, Cambridge, C.P., South Africa. |
| WEDDELL, C. W. .. .. .. .. .. .. .. .. | Boydville, 46 Miller Street, West Melbourne, Australia. |
| *WHITE, E. B. C. .. .. .. .. .. .. .. .. | Mental Hospital, Fishponds, Bristol. |
| WHITEHOUSE, H. BECKWITH, M.S. Lond. .. | 62 Hagley Road, Edgbaston, Birmingham. |
| *WIGODER, SYLVIA BEATRICE, M.A., M.D., Ph.D. | Radium Centre, Royal Infirmary, Bristol 1. |
| *WILBOND, R. G. .. .. .. .. .. .. .. .. | 92 Chesterfield Road, St. Andrew's, Bristol. |
| *WILLIAMS, I., M.B., Ch.B. Brist. .. .. .. | "Ardrem," Hill View, Henleaze, Bristol. |
| WILLIAMS, S. R., M.B. Lond. .. .. .. .. | Buckfastleigh, Devon. |
| *WILLS, W. K., *O.B.E.*, M.B., B.C. Cantab. .. | 1 Victoria Square, Clifton, Bristol 8. |
| *WOOD, DUNCAN .. .. .. .. .. .. .. .. | 105 Pembroke Road, Clifton, Bristol 8. |
| *WOOD, W. V., *M.C.*, B.A. Cantab. .. .. .. | Henley Lodge, Yatton, Som. |
| *WOODMAN, DOROTHY, M.D. Lond. .. .. .. | Kynance, Downs Cote Avenue, Westbury-on-Trym, Bristol. |
| *WOOLLEY, A. W., M.B., Ch.B. Brist. .. .. | The Royal Infirmary, Bristol 1. |
| *WOOLLEY, W., M.B., Ch.B... .. .. .. .. | 239 Cranbrook Road, Redland. |
| *WORTHINGTON, Lieut.-Col. F. .. .. .. .. | 1 Harley Place, Clifton, Bristol 8. |
| *WRIGHT, A. J., M.B., B.S. Lond. .. .. .. | 2 Clifton Park, Clifton, Bristol 8. |
| *WRIGHT, WINIFRED M., M.B., B.S. Lond. .. | 2 Waterloo Road, Shepton Mallet, Som. |
| *ZEALAND, L., M.D. Man. .. .. .. .. .. | Brecon Lodge, Westbury-on-Trym, Bristol. |

